# Does everybody with mildly elevated HsTnT without ECG changes have a high risk of cardiovascular events and mortality?

**DOI:** 10.1016/j.ijcha.2021.100838

**Published:** 2021-07-14

**Authors:** Yasar Sattar, Talal Almas, Monil Majmundar, Waqas Ullah, M. Chadi Alraies

**Affiliations:** Internal Medicine, Icahn School of Medicine at Mount Sinai, Queens, NY, USA; Internal Medicine, Royal College of Surgeons in Ireland, Dublin, Ireland; Internal Medicine, New York Medical College Metropolitan Hospital, New York, NY, USA; Internal Medicine, Abington Jefferson Health, PA, USA; Cardiology Department, Wayne State University, Detroit, MI, USA

Letter to Editor:

With interest we read the study by Mahmoud et al. about “ Comparative outcome analysis of stable mildly elevated high sensitivity troponin T in patients presenting with chest pain” [1]. The author reported higher frequency of all-cause and cardiovascular mortality up to 1 year in patients with low level hsTnT elevation.

A shortcoming of this study is that the author mentioned the cohort had normal or non-ischemic EKG with hsTNT levels below 50 ng/l; this statement implies a Type 2 MI or demand ischemia. Given the vast majority of this population do not receive coronary angiography during hospitalization, or elective or loss of follow up, it is imperative that the author mention all modifiable risk factors in baseline characteristics including A1C, and LDL level. Furthermore the author mentioned HsTNT < 99 percentile with level < 51 has the highest event rate at 1 year on KM analysis, it would be imperative to give the statistical cohort data based on age. Do all the patients over age > 18, including adult, middle age, and old age (octogenarian, nonagenarian, centenarian and supercentenarian), have the same risk of likely demand ischemia with even minor HsTNT elevation with no ischemic changes?. It would also be helpful to comment on how many people had sepsis on ED presentation since sepsis accounts for a significant portion of demand ischemia (HsTnT with no EKG changes).

It is equally imperative to note that in their study, the authors report meagre 30-day adverse cardiac event (ACE) rates of 0.1%, 0.6% and 0.4% for the groups 1, 2, and 3, respectively [1]. Due to the very low adverse event and mortality rates, the data cannot be extrapolated and generalisations about the underlying relation between low-level hsTnT elevation and mortality and ACE rates cannot reliably be drawn. Furthermore, the authors report a retrospective study involving merely 542 patients with low-level hsTn elevation [1]. Due to this limited sample size, reliable correlation between mildly elevated HsTnT levels and all-cause mortality cannot be drawn.

It should also be borne in mind that while hsTnT has often been touted as an effective indicator of future cardiac adverse events (ACEs) and all-cause, its use is fraught with uncertainties. For instance, high-sensitivity troponin T (HsTnT) as a diagnostic tool in patients with chest pain who present to the emergency department has a high rate of false-positive test results [2]. In this regard, the current study does not account for the possibility of false-positive results.

While this study sheds light on a focal component of triaging for cardiac adverse events, it has several limitations. The study could benefit from taking into account the aforementioned propositions so that conclusions pertaining to the diagnostic utility of hsTnT can reliably be drawn.

## Declaration of Competing Interest

The authors report no relationships that could be construed as a conflict of interest.
